# Research Progress on the Mechanism of Ginsenosides in the Treatment of Parkinson’s Disease

**DOI:** 10.3390/ijms27104544

**Published:** 2026-05-19

**Authors:** Shanshan Wang, Zhi Chen, Haipeng Tang, Jiyu Gong, Kejin Xu, Kangyu Wang

**Affiliations:** School of Pharmaceutical Sciences, Changchun University of Chinese Medicine, Changchun 130117, China; 13804343818@163.com (S.W.); cZ31678571661102@163.com (Z.C.); 17347074313@163.com (H.T.); gjy0431@126.com (J.G.)

**Keywords:** Parkinson’s disease, ginsenosides, neuroprotection, ferroptosis, multi-target intervention, oxidative stress, panax ginseng, protopanaxadiol, protopanaxatriol, α-synuclein

## Abstract

Parkinson’s disease (PD) is a neurodegenerative disorder of the central nervous system with a complex pathogenesis. Current conventional medicines are predominantly symptomatic treatments, which fail to reverse neuronal degeneration and often induce severe motor complications following long-term administration. In this context, the advantages of the multi-target holistic regulation provided by traditional Chinese medicine have become increasingly prominent. As the core active ingredients of *Panax ginseng*, ginsenosides can penetrate the blood–brain barrier and exhibit broad neuroprotective prospects in PD treatment. This article systematically reviews the neuroprotective mechanisms of different configurations of ginsenosides—mainly including protopanaxadiol (PPD) and protopanaxatriol (PPT) saponins—against PD. Studies indicate that PPD-type saponins (e.g., Rb1, Rg3, Rd) excel in directly inhibiting the abnormal aggregation of α-synuclein (α-syn), reducing oxidative stress, and preventing neuronal apoptosis. Conversely, PPT-type saponins (e.g., Rg1, Re) demonstrate significant advantages in suppressing microglia-mediated neuroinflammation, improving mitophagy, and regulating lipid metabolism networks. Furthermore, this review highlights a novel intervention strategy utilizing ginsenosides based on antioxidation and iron metabolism regulation. By maintaining the homeostasis of iron transport proteins such as DMT1 (Divalent Metal Transporter 1) and FPN1 (Ferroportin 1), and activating the Nrf2/xCT/GPX4 signaling axis, these compounds effectively block the vicious cycle of “iron deposition-oxidative stress-lipid peroxidation (LPO),” thereby inhibiting ferroptosis in dopaminergic neurons. In summary, structurally diverse ginsenosides exhibit distinct characteristics in targeting the core pathological events of PD. The scientific combination of ginsenoside monomers with different mechanisms in the future holds promise for constructing a comprehensive multi-target neuroprotective network, providing a solid theoretical foundation for novel ginsenoside-based combination therapies against PD.

## 1. Introduction

PD is a central neurodegenerative disorder characterized pathologically by the loss of dopaminergic neurons in the substantia nigra. Its clinical manifestations primarily include motor symptoms such as resting tremor, muscle rigidity, and bradykinesia, as well as non-motor symptoms including depression, dementia, and autonomic dysfunction [1,2]. According to the 2025 China Parkinson’s Disease Report, the current number of PD patients in China exceeds 5 million, accounting for approximately 43.14% of the global total. Furthermore, PD-related deaths in China represent about 23.71% of the global mortality. With the accelerating aging population, the number of PD patients is increasing and showing a trend toward younger onset. It is projected that by 2050, the number of patients in China will reach 10.5 million, ranking first in the world. Currently, the pathogenesis of PD involves multiple mechanisms, including abnormal aggregation of α-syn, oxidative stress, neuroinflammation, ferroptosis, and gut microbiota dysbiosis [3,4,5]. Due to the complexity of its pathogenesis, the exact mechanisms underlying Parkinson’s disease remain unclear, and a curative treatment is still lacking in clinical practice. Moreover, the disease is characterized by high disability rates and a prolonged clinical course. Long-term clinical follow-up studies have shown that most patients develop severe disability within 10 to 15 years of disease onset, becoming highly dependent on others for activities of daily living [6]. Without effective treatment and management, patients will experience a significantly shortened lifespan, leading to severe consequences for their life and health.

Currently, clinical management primarily relies on symptomatic treatment with Western medications such as levodopa. Although these drugs can ameliorate symptoms in the short term, long-term administration often leads to diminished efficacy and motor complications, including the “wearing-off” phenomenon and dyskinesias, as well as psychiatric disturbances [7]. In this context, the holistic therapeutic advantages of Traditional Chinese Medicine (TCM) have become increasingly prominent. In TCM theory, PD is classified under the categories of “tremor” and “spasm disease [8], its fundamental pathogenesis is characterized by “root deficiency and branch excess” [9]. The root deficiency manifests as Yin deficiency of the spleen and kidney, as well as deficiency of both Qi and blood, while the branch excess involves the internal generation of wind, fire, and turbid phlegm. These pathological factors interact with each other, creating a vicious cycle. Through syndrome differentiation and treatment, Chinese herbal medicine employs methods such as nourishing the liver and kidney, and supplementing Qi and nourishing blood. This approach enables multi-target holistic regulation and significantly reduces the toxic side effects of Western medications, thereby achieving the goal of addressing both the root cause and the manifestations [10]. Previous studies have found that Renshen Yangrong Tang (Ginseng Nourishing and Rejuvenating Decoction) can treat tremor syndrome caused by deficiency of both Qi and blood [11], Ginseng, as the sovereign herb in Ginseng Nourishing and Rejuvenating Decoction, is considered to be the active ingredient responsible for its therapeutic effects in treating tremor syndrome [12], this provides a theoretical basis for modern research to investigate the therapeutic mechanisms of ginseng in the treatment of PD.

Ginseng, the dried root and rhizome of *Panax ginseng* C.A. Mey. (Araliaceae), is characterized in Traditional Chinese Medicine theory as warm in property, sweet and slightly bitter in taste, and attributive to the spleen, lung, heart, and kidney meridians. The Shennong Bencao Jing (The Classic of Herbal Medicine) records that ginseng “mainly supplements the five viscera, calms the spirit, and with long-term use, promotes lightness of the body and prolongs life.” Modern research has also demonstrated that ginseng exerts extensive pharmacological effects on various conditions affecting the central nervous system and cardiovascular system. It exhibits properties such as enhancing learning and memory, anti-shock effects, anti-myocardial ischemia effects, and antioxidant activities, demonstrating unique biological activities and medicinal value [13]. The primary chemical constituents of ginseng include polysaccharides, ginsenosides, volatile oils, and organic acids. Among these, ginsenosides, as the main active components, are commonly considered to be the principal constituents responsible for its therapeutic effects in the treatment of PD [14]. Ginsenosides are primarily classified based on their aglycone structures into PPD type (e.g., Rb1, Rd, Rg3, CK), PPT type (e.g., Re, Rg1, Rg2), and oleanolic acid type (e.g., R3, Ro, and R4). This review summarizes the protective effects of various ginsenoside monomers against Parkinson’s disease, a central nervous system disorder, through multi-target and multi-pathway mechanisms, aiming to provide a reference for the clinical treatment of the disease.

## 2. Pathogenic Mechanisms of Parkinson’s Disease

### 2.1. Misfolding and Aggregation of α-Synuclein

α-syn is a core protein in the pathogenesis of PD, and its misfolding or abnormal aggregation into oligomers is considered a key factor contributing to neurotoxicity and neuronal death [15]. Yang et al. found that α-syn, as a naturally unfolded protein, can lead to mitochondrial damage, endoplasmic reticulum stress, and synaptic dysfunction when misfolded in neurons, resulting in severe injury to neurons and other cells [16]. Xu et al. found that when α-syn exhibits abnormal aggregation to form amyloid fibril structures, it spreads through the nervous system in a prion-like manner. This abnormal aggregation can lead to dual dysfunction of both presynaptic and postsynaptic functions, resulting in loss of synaptic transmission, abnormal synaptic plasticity, and dysregulation of neuronal activity, ultimately leading to neuronal death [17]. Jeswinder Sian-Hulsmann and Michael X. Henderson observed that initial small aggregates of α-syn appear in the neuronal cytoplasm, subsequently forming a classical Lewy body [18,19]. Lewy bodies are space-occupying lesions that are harmful to neurons, interfering with normal intracellular functions such as mitochondrial function, the autophagy-lysosome pathway, and the ubiquitin-proteasome system. This disruption leads to cellular dysfunction and loss, ultimately contributing to the pathogenesis of Parkinson’s disease [20].

### 2.2. Oxidative Stress and Dysregulation of Metal Ion Metabolism

Recent studies have suggested that oxidative stress is closely associated with PD and is considered one of the important factors contributing to the death of dopaminergic neurons [21]. Oxidative stress is a pathological process resulting from an imbalance between the oxidative and antioxidant systems in the body, where the production and scavenging of free radicals become disrupted. This leads to excessive accumulation of reactive oxygen species (ROS) and peroxidation products, ultimately causing cellular damage and even death [22]. Studies have found that excessive production of reactive oxygen species directly attacks lipids, proteins, and nucleic acids, thereby impairing cellular structure and function [23]. Pfeifer GP reported that elevated ROS levels induced by oxidative stress can cause DNA damage, which slows or blocks the progression of RNA polymerase II, leading to transcriptional stress and subsequent mutations, ultimately resulting in loss of neuronal integrity and cell death [24]. Zhang et al. found that the autoxidation of dopaminergic neurons in the substantia nigra generates a large amount of iron ions. These reduced iron ions can react with hydrogen peroxide produced during dopamine metabolism, thereby generating highly toxic hydroxyl radicals. This subsequently induces LPO, leading to apoptosis of nigral neurons and the manifestation of PD symptoms [25].

Dysregulation of metal ion metabolism is also one of the pathogenic mechanisms of PD, including ferroptosis and cuproptosis. Ferroptosis is a form of non-apoptotic cell death, defined as iron-dependent regulatory cell death caused by membrane damage mediated by extensive LPO [26]. Studies have found that abnormal iron accumulation can lead to decreased dopamine levels in the striatum, promoting the development of motor deficits in PD [27]. Simultaneously, α-syn present in cells incorporates into the cell membrane in an iron-dependent manner, promoting cellular LPO. This leads to increased production of ROS, accelerates oxidative stress, and ultimately induces ferroptosis [28]. Studies [29] have also found that MRI (Magnetic Resonance Imaging) of the brain in PD patients reveals significantly increased iron content in the substantia nigra and globus pallidus, along with a specific decrease in glutathione (GSH) levels. Additionally, abnormally elevated levels of LPO have been detected in the brain tissue of deceased PD patients. These findings represent key characteristics of the ferroptosis-mediated cell death pathway.

### 2.3. Mitophagy

Previous studies have shown that abnormal mitophagy leading to the death of dopaminergic neurons is one of the mechanisms underlying early-onset PD. Balancing the mitophagy process is crucial for maintaining neuronal homeostasis [30]. Mitophagy is a selective autophagic process that removes damaged or unwanted mitochondria to maintain cellular and organismal homeostasis [31]. Yu et al. found that mitophagy selectively eliminates damaged mitochondria through the PTEN-induced kinase 1 (PINK1)/Parkin pathway, thereby maintaining energy homeostasis. In PD mutations in the PINK1 gene (such as G411S) impair mitophagy, which subsequently promotes ROS accumulation, activates the c-Jun N-terminal kinase (JNK)/p53 pathway, and induces neuronal apoptosis [32]. Li et al. found that when mitochondrial transport is impaired, mitophagy is aberrantly activated in an attempt to ameliorate local mitochondrial dysfunction and reduce oxidative stress caused by excessive ROS. However, this abnormal activation ultimately leads to mitochondrial dysfunction and neuronal cell death [33].

### 2.4. Neuroinflammation and Immune Response

Accumulating evidence suggests that neuroinflammation plays a significant role in the pathogenesis and progression of PD. Microglia, as the primary immune effector cells in the central nervous system, are critically involved. Persistent neuroinflammation resulting from their abnormal activation is considered a key mechanism driving the degeneration of dopaminergic neurons [34,35]. Activated microglia release pro-inflammatory cytokines such as interleukin-1β (IL-1β), interleukin-6 (IL-6), and tumor necrosis factor-α (TNF-α), thereby forming a neurotoxic microenvironment. These inflammatory mediators not only directly damage neurons but also further activate surrounding glial cells through positive feedback mechanisms [36]. Effective inhibition of neuroinflammation may help slow disease progression. Therefore, exploring novel therapies that can modulate neuroinflammation has become a research hotspot [37].

### 2.5. Lipid Metabolism Regulation

In recent years, research on the relationship between lipid metabolism and PD has been increasing, revealing that lipid metabolism disorders play a critical role in the pathological mechanisms of PD [38]. Studies have revealed that abnormal accumulation of lipid droplets in neurons may promote the conversion of α-syn into a form resistant to proteolysis, thereby leading to its aggregation in human neurons. This phenomenon suggests that abnormal lipid metabolism may play an important role in the pathogenesis of PD [39]. Kai Zhang and Zhang-Li Wang found that the key regulators of lipid metabolism are unsaturated fatty acids and lipid hydroperoxides (LOOH). Dysregulation in any part of this metabolic regulatory mechanism can induce LPO, ultimately leading to damage of dopaminergic neurons [40,41]. Lu et al. found that lipids are involved in many aspects of PD pathology, including specific cytotoxic interactions with α-syn, increased risk of PD associated with mutations in genes encoding lipid metabolism-related enzymes, alterations in lipid pathways, and the participation of lipids in oxidative stress and inflammation. Therefore, abnormal lipid metabolism is considered a potential risk factor for the development of PD [42].

### 2.6. Gut Microbiota

Studies have suggested that the pathogenesis of PD may originate in the gastrointestinal tract, and the relationship between gastrointestinal function and PD has become a research hotspot in recent years. It has been reported that approximately 80% of PD patients experience gastrointestinal dysfunction, particularly constipation [43]. One of the main pathological features of PD is the abnormal aggregation of α-syn. Studies have found that α-syn not only regulates neurotransmission in the brain but also modulates gastrointestinal function. α-Syn protein can be formed in the gut and spread from the gut to the brain, supporting the hypothesis that the pathogenesis of PD may be mediated through the intestinal tract [44]. Gut microbiota dysbiosis may also exacerbate the pathological progression of PD by affecting mechanisms such as intestinal barrier function, immune response, and neuroinflammation [45]. Zhe Zhao et al. found that gut microbiota imbalance may lead to neuroinflammation and neurodegeneration. Mice transplanted with microbiota from PD patients exhibited more severe motor dysfunction and greater loss of dopaminergic neurons compared to mice transplanted with microbiota from healthy subjects, suggesting that dysbiosis is a risk factor for PD [46].

## 3. Multi-Target Neuroprotective Mechanisms of Different Types of Ginsenosides in PD

**Figure 1:**
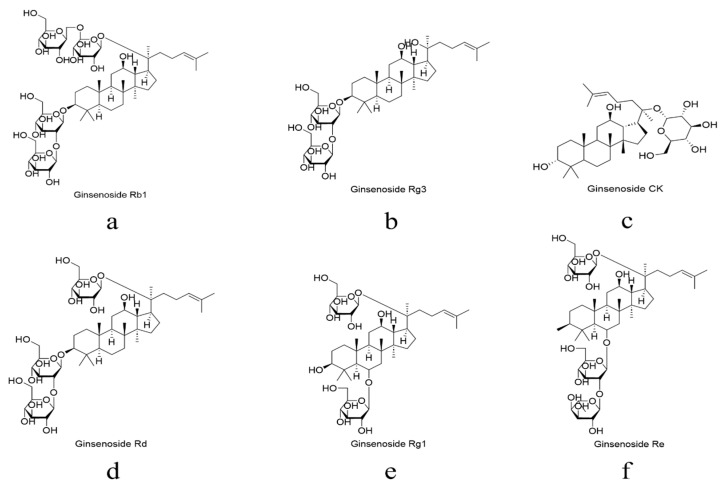
Chemical structures of the principal ginsenoside monomers discussed in this review. Protopanaxadiol (PPD)-type ginsenosides include (**a**–**d**), while protopanaxatriol (PPT)-type ginsenosides include (**e**,**f**).

To better understand the structure-activity relationships and the pharmacological basis discussed in the subsequent sections, the specific chemical structures of the representative PPD-type and PPT-type ginsenoside monomers are presented in Figure 1.

**Figure 2:**
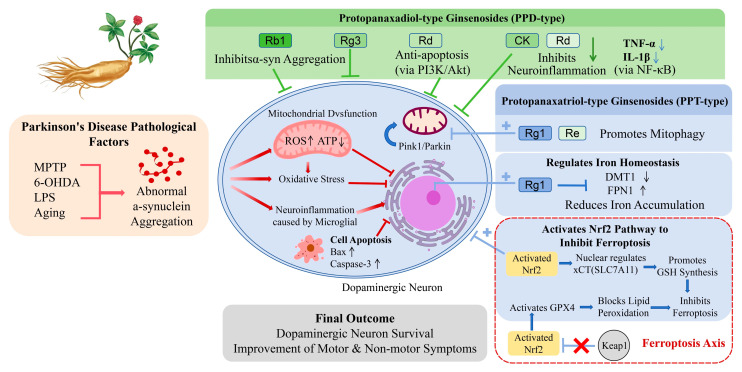
Schematic diagram of the multi-target neuroprotective mechanisms of ginsenosides in PD. Pathological triggers induce abnormal α-synuclein aggregation, driving a cascade of mitochondrial dysfunction, oxidative stress, neuroinflammation, and neuronal apoptosis (indicated by red arrows). Ginsenosides exert synergistic neuroprotective effects depending on their structures. Protopanaxadiol (PPD)-type ginsenosides primarily inhibit protein aggregation, neuroinflammation, and apoptosis (indicated by green inhibitory T-bars). Conversely, protopanaxatriol (PPT)-type ginsenosides prominently promote mitophagy, regulate iron homeostasis, and activate antioxidant defenses (indicated by blue arrows with plus signs +). Notably, the dissociation of Nrf2 from Keap1 (indicated by the red cross ×) upregulates xCT and glutathione (GSH) synthesis, thereby blocking lipid peroxidation and ferroptosis. Abbreviations: PD, Parkinson’s disease; PPD, protopanaxadiol; PPT, protopanaxatriol; α-syn, α-synuclein; ROS, reactive oxygen species; ATP, adenosine triphosphate; TNF-α, tumor necrosis factor-alpha; IL-1β, interleukin-1 beta; DMT1, divalent metal transporter 1; FPN1, ferroportin 1; Keap1, Kelch-like ECH-associated protein 1; Nrf2, nuclear factor erythroid 2-related factor 2; xCT, cystine/glutamate transporter; GSH, glutathione; GPX4, glutathione peroxidase 4.

As the core active components of *Panax ginseng*, ginsenosides are capable of crossing the blood–brain barrier and exerting multi-target effects against the complex pathological processes of PD [47,48]. As illustrated in Figure 2, these monomers—classified into PPD and PPT types—synergistically protect dopaminergic (DA) neurons through a comprehensive network. Existing studies indicate that their primary mechanisms include inhibiting α-synuclein aggregation, alleviating mitochondrial dysfunction and oxidative stress [49,50], suppressing neuroinflammation [51,52], preventing neuronal apoptosis [53], and regulating iron metabolism to block ferroptosis [54].

### 3.1. Protopanaxadiol Type Ginsenosides

#### 3.1.1. Ginsenoside Rb1

Rb1 represents a promising PPD-type ginsenoside with potent multi-target effects against the core pathologies of PD, specifically protein aggregation, neuroinflammation, and oxidative stress (Table 1). Unlike many other saponins, Rb1 can directly intervene in protein pathology by inhibiting α-syn fibrillation and disaggregating preformed fibrils [55]. Its anti-inflammatory efficacy is largely mediated by the inhibition of microglial activation and the subsequent release of pro-inflammatory mediators via the NF-κB signaling pathway [56,57]. Furthermore, Rb1 enhances endogenous antioxidant defenses by activating the Gβ1/PI3K/Akt-Nrf2 axis [49], and its anti-apoptotic properties involve the regulation of PI3K/Akt, MAPK, and Notch signaling pathways [58,59].

#### 3.1.2. Ginsenoside Rg3

Rg3 primarily exerts neuroprotective effects by bolstering antioxidant defenses and inhibiting neuronal apoptosis. It significantly elevates the expression of Nrf2 and its downstream antioxidant enzymes, such as HO-1, NQO1, and glutamate-cysteine ligase (GCLC/GCLM), thereby effectively scavenging ROS in the nigrostriatal system [60,61]. In addition to its antioxidant role, Rg3 suppresses the caspase cascade (e.g., inhibiting EGL-1 and CED-3) to prevent the loss of dopaminergic neurons, showcasing its potential as a dual-action neuroprotective agent [69].

#### 3.1.3. Ginsenoside CK and Ginsenoside Rd

Both CK and Rd demonstrate significant potential in modulating the neuronal microenvironment. CK specifically targets neuroinflammation by suppressing pro-inflammatory cytokines (IL-1β, IL-6, TNF-α) while promoting anti-inflammatory IL-2 expression [70]. Meanwhile, Rd focuses on mitochondrial preservation, stabilizing membrane potential and maintaining respiratory complex I activity through the PI3K/Akt pathway, which ultimately restores intracellular ATP levels under oxidative stress [71,72].

### 3.2. Protopanaxatriol Type Ginsenosides

#### 3.2.1. Ginsenoside Rg1

Rg1 is perhaps the most extensively studied PPT-type ginsenoside, demonstrating a comprehensive neuroprotective profile that bridges central and peripheral immunity while regulating iron homeostasis. It modulates peripheral T cell subsets (e.g., increasing Treg cells) and inhibits central microglial polarization towards a pro-inflammatory M1 phenotype [73,74]. These anti-inflammatory effects are further supported by the suppression of NF-κB, JNK, and p-c-Jun/COX-2 signaling [75,76]. Beyond immunity, Rg1 is a critical regulator of brain iron metabolism; it reduces nigral iron overload by downregulating the importer DMT1 and upregulating the exporter FPN1 [54,77,78,79]. Additionally, Rg1 alleviates endoplasmic reticulum stress and enhances dopamine synthesis through EphA5 signaling [80,81,82].

#### 3.2.2. Ginsenoside Re

Ginsenoside Re excels in restoring mitochondrial quality control and lipid metabolic balance. It activates the PINK1/Parkin-mediated mitophagy pathway to clear damaged mitochondria and modulates mitochondrial dynamics via Marf and Drp1 [83,84]. Complementary to its mitochondrial protection, Re activates the Nrf2-mediated antioxidant program [50] and disrupts the vicious cycle of lipid peroxidation by regulating sphingolipid and arachidonic acid metabolism [78,79,80].

The synergistic potential of PPT-type saponins is further evidenced by studies showing that the combination of Re and Rd provides superior protection against mitochondrial impairment compared to single-agent treatments [81].

### 3.3. Comparative Efficacy and Evidence Strength of PPD- and PPT-Type Ginsenosides

While the aforementioned studies outline the diverse mechanisms of various ginsenosides, a critical evaluation reveals significant disparities in their evidence strength and translational potential. Currently, the evidence supporting Rb1 and Rg1 is the most robust, primarily derived from extensive in vivo studies using classic PD rodent models (e.g., MPTP and 6-OHDA) [1,54]. In contrast, the evidence for Re and Rd remains largely confined to in vitro cellular models (such as SH-SY5Y cells) [64,81]. Although these minor ginsenosides show promising antioxidant properties, their in vivo neuroprotective efficacy lacks sufficient validation. Furthermore, a major limitation in the current literature is the absence of head-to-head comparative studies evaluating these monomers under identical experimental conditions. Consequently, while theoretical mechanisms are well-documented, it remains challenging to definitively rank their absolute therapeutic potency, highlighting a critical gap that future comparative pharmacological studies must address.

## 4. Ferroptosis as a Central Therapeutic Target and the Integrated Multi-Target Network of Ginsenosides

In the pathological progression of PD, individual mechanisms such as α-synuclein aggregation, impaired mitophagy, and iron dyshomeostasis form a cohesive “vicious cycle” rather than acting in isolation. Specifically, misfolded α-synuclein and damaged mitochondria serve as the primary triggers, significantly elevating intracellular reactive oxygen species (ROS) levels. This oxidative environment promotes abnormal accumulation of iron ions, driving the Fenton reaction to generate substantial hydroxyl radicals, which subsequently induce extensive lipid peroxidation (LPO), ultimately leading to iron-dependent programmed cell death—ferroptosis [27,28]. Ginsenosides exert a unique “integrated neuroprotective effect” by disrupting this cascade at multiple nodes. Beyond their traditional antioxidant roles, they stabilize iron proteostasis and activate the Nrf2/GPX4 axis, thereby providing a robust defense network that simultaneously addresses protein pathology and iron-dependent lipid damage.

### 4.1. Disrupting the “Iron-Oxidative Stress” Vicious Cycle

Studies have found that increased levels of free iron ions not only directly exacerbate oxidative damage but also promote the aggregation and fibrillation of α-syn, which in turn further disrupts cell membrane integrity [28]. Targeting this mechanism, ginsenoside Rg1 has demonstrated specific intervention potential. Wang et al. [54] confirmed that Rg1 effectively reduces iron levels in the substantia nigra of MPTP-induced PD model mice. The mechanism involves the regulation of specific iron transport proteins (such as DMT1 and FPN1), thereby reducing neuronal iron overload. By decreasing the labile iron pool within cells, Rg1 inhibits the occurrence of the Fenton reaction at its source and reduces the generation of highly toxic hydroxyl radicals.

### 4.2. Inhibition of Lipid Peroxidation and Core Ferroptosis Pathways

**Figure 3:**
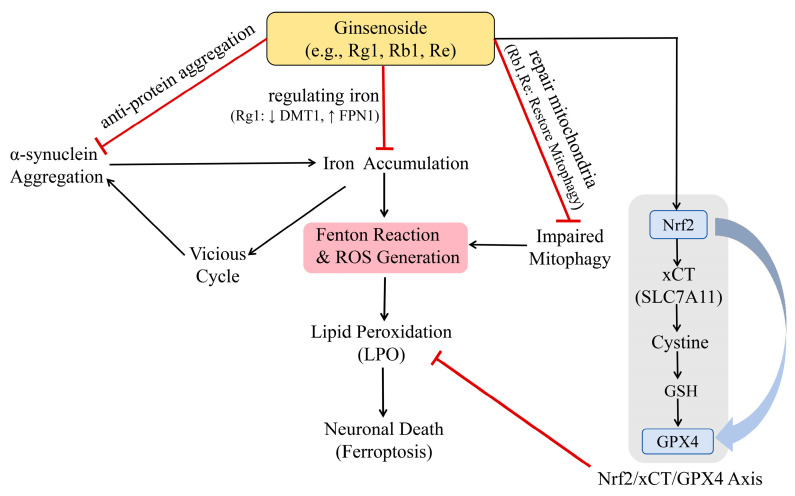
The multi-target regulatory network of ginsenosides against the iron-dependent ferroptosis cascade in Parkinson’s disease. α-synuclein aggregation, iron accumulation, and impaired mitophagy synergistically drive ROS generation and LPO (indicated by black arrows), forming a pathogenic vicious cycle that leads to ferroptotic neuronal death. Ginsenosides disrupt this network by: (1) inhibiting α-synuclein aggregation; (2) mitigating iron overload via regulating transporters (DMT1/FPN1); and (3) restoring mitophagy (indicated by red T-bars). Furthermore, ginsenosides activate the Nrf2/xCT/GPX4 signaling axis (indicated by thick blue arrows) to enhance GSH synthesis and directly clear toxic lipid peroxides, thereby blocking ferroptosis. Abbreviations: DMT1, divalent metal transporter 1; FPN1, ferroportin 1; ROS, reactive oxygen species; LPO, lipid peroxidation; Nrf2, nuclear factor erythroid 2-related factor 2; xCT (SLC7A11), cystine/glutamate transporter; GSH, glutathione; GPX4, glutathione peroxidase 4.

To counteract the downstream effects of this cascade reaction, the key target of ginsenosides lies in activating the Nrf2/xCT/GPX4 signaling axis. This pathway maintains cystine uptake and promotes glutathione synthesis, thereby enhancing the repair capacity of membrane lipid peroxides and ultimately blocking the execution of ferroptosis. The central role of this Nrf2-mediated mechanism in inhibiting lipid peroxidation and preventing dopaminergic neuronal loss is systematically illustrated in Figure 3.

#### 4.2.1. Activation of the Nrf2/xCT/GPX4 Signaling Axis

The core mechanism by which ginsenosides inhibit ferroptosis involves the upregulation of nuclear factor erythroid 2-related factor 2 (Nrf2) and its downstream target genes xCT and glutathione peroxidase 4 (GPX4). Nrf2 serves as the master switch of cellular antioxidant defense and is normally sequestered in an inactive state bound to Keap1. Ginsenosides (such as Rg1, Re, and Rb1) promote the dissociation of Nrf2 from Keap1 and its translocation into the nucleus, thereby initiating downstream gene transcription. xCT (SLC7A11), the light chain subunit of the cystine/glutamate antiporter, is responsible for cystine uptake for GSH synthesis. By upregulating xCT, ginsenosides maintain intracellular GSH levels, providing the essential substrate for GPX4. GPX4 is a key negative regulator of ferroptosis that reduces toxic lipid peroxides in cell membranes to nontoxic lipid alcohols. Ginsenosides Re and Rg1 have been shown to significantly restore GPX4 expression under pathological conditions, thereby directly interrupting the LPO chain reaction [82,83].

However, it is crucial to distinguish between general antioxidation and specific ferroptosis regulation. While numerous studies demonstrate the robust ROS-scavenging abilities of ginsenosides, direct experimental evidence explicitly confirming their modulation of core ferroptotic markers (e.g., GPX4 activity or lipid peroxide clearance) in PD models remains limited. Current inferences are largely drawn from their performance in related neurodegenerative or ischemic models, highlighting a critical direction for future targeted validation.

#### 4.2.2. Advantages and Prospects of Multi-Target Combination Intervention

Compared to single-target iron chelators (such as deferiprone), ginsenosides exhibit a comprehensive “multi-component, multi-target” advantage. They not only reduce brain iron burden similarly to iron chelators (primarily through Rg1) but also concurrently repair mitochondrial function, scavenge free radicals, and regulate lipid metabolism (primarily through Rb1 and Re). This synergistic mode of action precisely addresses the complex pathological mechanisms of PD by simultaneously intervening in the three key processes of ferroptosis: dysregulated iron metabolism, oxidative stress, and LPO. Future research should further elucidate the specific molecular targets through which ginsenosides regulate iron transport proteins and explore their potential to enhance efficacy and reduce toxicity when combined with levodopa.

To provide a clearer overview of the current research landscape, a comprehensive summary of the specific molecular mechanisms, corresponding biological targets, and experimental models associated with individual ginsenoside monomers in PD is systematically outlined in Table 1.

## 5. Clinical Translation, Safety, and Therapeutic Challenges

Despite the overwhelmingly positive preclinical data, the translation of ginsenosides into clinical PD therapeutics faces significant challenges that must be addressed.

### 5.1. Pharmacokinetics and BBB Penetration

A primary obstacle is their poor pharmacokinetic profile. Most ginsenoside monomers (such as Rb1 and Re) exhibit remarkably low oral bioavailability (<5%) and limited ability to penetrate the blood–brain barrier (BBB) due to their high polarity and large molecular weight [47,48]. Future research must urgently explore nano-delivery systems, such as exosomes or lipid nanoparticles, to enhance central nervous system accumulation and therapeutic efficacy [84].

### 5.2. Clinical Trials and Toxicity

Currently, rigorous, randomized, double-blind clinical trials evaluating highly purified ginsenoside monomers in human PD patients are lacking. Most clinical evidence relies on unstandardized crude *Panax ginseng* extracts, leading to inconsistent results [85]. Regarding safety, while ginsenosides are generally well-tolerated with low systemic toxicity in acute models, comprehensive long-term toxicological data in elderly populations are required to establish a safe therapeutic window.

### 5.3. Drug Interactions with Levodopa

Furthermore, potential interactions with conventional PD medications demand clinical attention. On a positive note, ginsenosides present a promising role as adjuvant therapies to delay or alleviate levodopa-induced dyskinesia (LID) through their multi-target neuroprotective networks [86]. However, because certain ginsenosides can modulate cytochrome P450 enzyme activity, careful dosage considerations are essential to avoid unpredictable metabolic interactions with L-dopa or other synthetic drugs [87].

## 6. Conclusions and Future Directions: Combination and Multi-Target Therapies

In summary, structurally diverse ginsenosides exhibit distinct advantages in intervening in PD. PPD-type ginsenosides (e.g., Rb1, Rg3) demonstrate excellent performance in directly counteracting core pathologies such as α-synuclein aggregation, while also providing robust antioxidant and anti-apoptotic effects. Conversely, PPT-type ginsenosides, particularly Rg1 and Re, exhibit comprehensive mechanisms in regulating iron metabolism disorders, restoring mitochondrial quality, and inhibiting neuroinflammation. Notably, ginsenoside Rg1 shows exceptional promise as a multi-target candidate specifically targeting ferroptosis. Its core potential lies in effectively regulating brain iron homeostasis, thereby blocking upstream oxidative stress and lipid peroxidation (LPO)—disease-modifying functions that current conventional medications largely lack. However, emerging studies demonstrate that the combined use of different ginsenosides produces powerful synergistic effects. A scientific formulation of ginsenosides with complementary mechanisms (such as combining Re with Rd, or integrating Rg1, Rb1, and Re) holds immense promise for establishing a multi-target therapeutic network. This strategy covers the entire “iron metabolism-oxidative stress-LPO-protein aggregation-neuroinflammation” pathological cascade, potentially achieving superior neuroprotective effects compared to single-component treatments. Ultimately, while significant limitations remain—particularly regarding their poor pharmacokinetic profiles, limited blood–brain barrier permeability, and the scarcity of human clinical trials—the continuous optimization of ginsenoside-based combination therapies provides a compelling future direction for fundamentally modifying the progression of Parkinson’s disease.

## Figures and Tables

**Table 1:** Summary of the molecular mechanisms and experimental models of ginsenoside monomers in Parkinson’s disease.

Ginsenoside	Mechanism (Shared Pathways)	Model	Key Findings	Evidence Level	References
Rb1	Anti-protein aggregation	Recombinant α-syn protein	Inhibits α-synuclein fibrillation/aggregation and disaggregates preformed fibrils.	In vitro	[55]
Rb1	Anti-neuroinflammation (NF-κB pathway)	LPS-induced rats; BV2 cells	Suppresses glial inflammatory response by inhibiting pro-inflammatory mediators via NF-κB.	In vivo & In vitro	[56]
Rb1	Antioxidant stress (PI3K/Akt-Nrf2)	6-OHDA-induced SH-SY5Y cells	Induces HO-1 through the Gβ1/PI3K/Akt-Nrf2 signaling pathway to protect cells.	In vitro	[49]
Rg3	Antioxidant stress (Nrf2/HO-1)	MPTP-induced mice; Rotenone-induced mice	Activates Nrf2/HO-1/NQO1 pathway; increases GCLC/GCLM; significantly reduces ROS in the substantia nigra.	In vivo	[60,61]
Rg1	Anti-neuroinflammation (NF-κB & COX-2)	MPTP/LPS-induced mice	Inhibits microglial activation, reduces TNF-α, IL-1β, IL-6; inhibits NF-κB nuclear translocation and p-c-Jun/COX-2.	In vivo	[62,63,64]
Rg1	Iron metabolism & Ferroptosis (DMT1/FPN1)	MPTP-induced mice; MPP^+^-induced MES23.5 cells	Downregulates iron importer DMT1 and upregulates exporter FPN1; alleviates iron-dependent oxidative stress.	In vivo & In vitro	[54,65,66,67]
Re	Mitophagy & Antioxidant (Parkin/Pink-1, Nrf2)	Rotenone-induced Drosophila/SH-SY5Y cells	Activates Parkin/Pink-1 to clear damaged mitochondria; activates Nrf2 to attenuate oxidative stress.	In vivo & In vitro	[50,68]

Table notes: MPTP, 1-Methyl-4-phenyl-1,2,3,6-tetrahydropyridine; α-syn, α-Synuclein; 6-OHDA, 6-Hydroxydopamine; LPS, Lipopolysaccharide; GCLC, Glutamate-Cysteine Ligase Catalytic Subunit; GCLM, Glutamate-Cysteine Ligase Modifier Subunit; IL-1β, Interleukin-1 Beta; TNF-α, Tumor Necrosis Factor-Alpha; HO-1, Heme oxygenase-1; NQO1, NAD(P)H quinone dehydrogenase 1; NF-κB, Nuclear factor kappa B; IL-6, Interleukin-6; MPP^+^, 1-Methyl-4-phenylpyridinium; ROS, Reactive Oxygen Species; COX-2, Cyclooxygenase-2; DMT1, Divalent Metal Transporter 1; FPN1,Ferroportin 1; Nrf2, Nuclear factor erythroid 2-related factor 2.

## Data Availability

No new data were created or analyzed in this study. Data sharing is not applicable to this article.
